# Effects of Heat Shock Protein 70 Gene Polymorphism on Heat Resistance in Beef and Dairy Calves Based on Proliferation and Heat Shock Protein 70 Gene Expression in Peripheral Blood Mononuclear Cells and Hair Follicles

**DOI:** 10.3390/ani15040475

**Published:** 2025-02-07

**Authors:** Won Seob Kim, Yong Ho Jo, Jalil Ghassemi Nejad, Hong Gu Lee

**Affiliations:** 1 Department of Animal Science and Technology, Sanghuh College of Life Sciences, Konkuk University, Seoul 05029, Republic of Korea; kws9285@konkuk.ac.kr (W.S.K.); jyh3977@naver.com (Y.H.J.); jalilghaseminejad@gmail.com (J.G.N.); 2 IANS Co., Ltd., Cheonan-si 31090, Republic of Korea; 3 GeneBiotech/DaOne Chemical, Seongnam-si 13105, Republic of Korea

**Keywords:** HSP70 genetic polymorphism, cattle, heat resistance, immune cells, hair follicles

## Abstract

The study objectives were to investigate the heat resistance using peripheral blood mononuclear cells (PBMCs) and hair follicles in beef and dairy calves based on heat shock protein (HSP) 70 genetic polymorphisms. The hair follicle samples from sixty calves (6 months old; 30 Korean native beef calves and 30 Holstein dairy calves) were collected for DNA extraction. The HSP70 single nucleotide polymorphism (SNP) was genotyped using a 5′-exonuclease activity (TaqMan) assay. In Study 1, PBMCs were isolated from 20 calves categorized by their HSP70 genotypes during a thermoneutral period: 10 Korean native beef calves (B-CC and B-C/-type) and 10 Holstein dairy calves (D-CC, D-C/-type). The PBMCs were then exposed to heat stress at 37 °C (control, CON) and 42 °C (heat stress, HS) for 3 h. Following this, the cells were returned to the 37 °C incubator at 0, 1, 3, 6, and 12 h for further recovery analysis. In Study 2, hair follicles were collected from 20 calves (six times every 30 days; threshold, mild, and moderate stress levels) and HSP70 gene expression was measured. Data were analyzed via two-way analysis of variance (ANOVA) and Tukey’s honestly significant difference (HSD) test. The cell proliferation in the D-C/-group was significantly higher (*p* < 0.05) than in the D-CC and B-C/-groups at 0 and 1 h after HS for 3 h. The mRNA gene expression of HSP70 was greater (*p* < 0.01) in all HS groups compared to the CON groups after heat exposure. The expression of the HSP70 gene in the D-C/-group was significantly higher (*p* < 0.05) compared to the B-CC and B-C/-groups immediately (0 h) following 3 h of HS. The expression in the D-CC group also higher (*p* < 0.05) than in the B-C/-group. The gene expression of HSP70 in hair follicles increased more at the moderate HS level than that at the threshold level. In addition, overexpression of HSP70 was noted (*p* < 0.05) in the D-CC and D-C/-groups compared to the B-CC and B-C/-groups. In conclusion, our results indicate that breeds and HSP70 genetic polymorphisms exhibit a distinctive pattern of immune cell proliferation and HSP70 expression profiles. Additionally, the HSP70 gene expression in hair follicles may serve as an indicator of heat resistance across different breeds, making it a potential novel barometer for HS.

## Simple Summary

Heat stress (HS) impacts cattle health, productivity, and immune functions, particularly in warm climates. In this study, we aimed to investigate genetic variations in the heat shock protein 70A (HSP70A) gene (with a deletion of cytosine at base position 895) and their impact on heat resistance in beef and dairy calves. HS reduced cell proliferation in peripheral blood mononuclear cells across all HSP70 genotype groups, with a greater impact observed in dairy calves than in beef calves. The expression of the HSP70 gene significantly increased during HS, particularly in dairy calves with certain genetic variants (C/-type), suggesting a stronger stress response than in beef calves. In addition, HSP70 expression in hair follicles increased with an increase in temperature–humidity index levels, making HSP70 expression a potential non-invasive indicator of heat tolerance. The results obtained in this study revealed that calf breeds (Korean native beef calves and Holstein dairy calves) and genetic variations in thermotolerance because of HSP70 polymorphisms affected cellular responses and HSP70 gene expression in response to HS. The findings suggest that HSP70A single nucleotide polymorphisms in cattle provide opportunities to enhance livestock resilience through genetic selection and management strategies to reduce HS.

## 1. Introduction

Heat stress (HS), driven by global warming, disrupts physiological mechanisms in cattle, causing metabolic disorders and weakened immune function [1]. Among ruminants, this phenomenon affects both the dairy and beef industries, incurring annual losses of approximately $ 1.2 billion and $ 369 million, respectively [2]. A promising approach to address these challenges is to enhance genetic programs to select thermotolerant animals, with improved cattle productivity and resilience under HS conditions [3,4].

Genetic variations that impact thermotolerance in cattle have been widely studied, focusing on cattle physiology and productivity [4,5,6]. Cellular thermotolerance under HS is predominantly regulated by heat shock proteins (HSPs) that play critical roles in maintaining cellular homeostasis at elevated temperatures [7]. Among these, HSP70 is the most abundant and highly temperature-sensitive [7,8], making it a key indicator of thermotolerance and a promising target for enhancing heat resilience in cattle. A previous study reported the potential of HSP70 polymorphisms in improving thermotolerance and productivity in cattle under HS conditions [9]. Similarly, associations between single nucleotide polymorphisms (SNPs) in HSP70 and various physiological and physical traits have been reported in Bali cattle [10]. At the cellular level, HS has been shown to induce the upregulation of HSP70 gene expression in peripheral blood mononuclear cells (PBMCs) and skeletal muscle cells, suggesting the critical role of HSP70 in adaptive response to HS [7,8].

In addition to HSP70 genotypes, differences in cattle breeds impacting metabolic mechanisms underlying milk and meat production play a significant role in determining thermotolerance in cattle [11]. Dairy cows are particularly susceptible to HS because of the substantial metabolic heat generated by their elevated milk production, which make them more susceptible to the adverse effects of thermal stress compared to other cattle breeds [12].

A previous study has suggested that HSP70 gene expression in hair follicles is a novel indicator of HS in cattle [13]. Previous studies have demonstrated that HSP70 gene expression in hair follicles rapidly increases under external HS conditions, focusing on its potential as a sensitive biomarker for assessing thermal stress in livestock [13]. However, to the best of the authors’ knowledge, the effects of different cattle breeds or HSP70 genotypes on HSP70 gene expression in hair follicles under HS conditions have not been evaluated.

Therefore, we hypothesized that differences between the breeds and HSP70 genotypes of beef and dairy calves may impact their thermal responses at the cellular level and HSP70 gene expression in their hair follicles in response to external environmental conditions. In this study, we aimed to evaluate the differential effects of different cattle breeds and HSP70 genotypes on the proliferation of PBMCs and the expression of HSP70 in PBMCs and hair follicles under HS conditions. Our findings provide information on key factors determining thermotolerance in cattle that must be considered in genetic programs aimed at improving cattle productivity and resilience under HS conditions.

## 2. Materials and Methods

**Figure 1:**
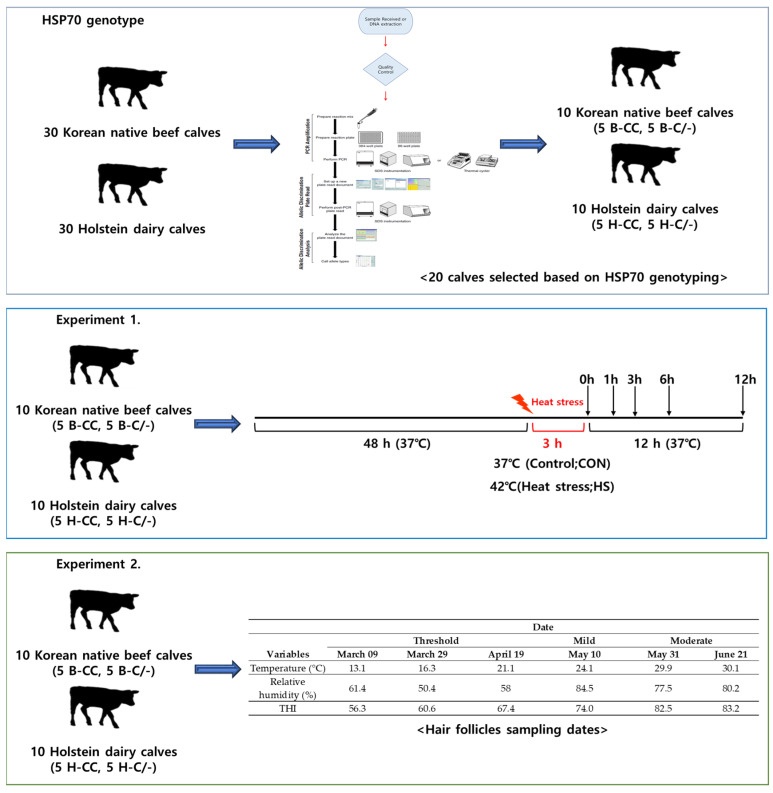
Experimental design schematic.

All the procedures involving animals were approved by the Institutional Animal Care and Use Committee (IACUC) of Konkuk University (Approval No: KU22151). Figure 1 illustrates the experimental design used to investigate the HSP70 genotype and the effects of HS on PBMCs and hair follicles.

### 2.1. HSP70 Genotype

#### 2.1.1. DNA Extraction from Hair Follicles for Determining HSP70 Genotype

Hair follicle samples were obtained from 60 calves (6 months old; 30 Korean native beef calves and 30 Holstein dairy calves). DNA was manually extracted from hair follicles in the tails [14]. Sections of 5–10 mm thickness were sliced from 20 hair follicles and transferred to a 1.5 mL tube. A total of 300 µL of tissue lysis (TL) buffer, containing 1 M Tris-Cl (pH 8.0), 0.5 M ethylenediaminetetraacetic acid (EDTA) (pH 8.0), 5 M NaCl, 10% sodium dodecyl sulfate, and distilled water, was added and mixed by inversion. Subsequently, 5 µL proteinase K (20 mg/mL, G-spin Total DNA extraction Kit, iNtRON Biotechnology, Seongnam, Republic of Korea) was added, and the mixture was incubated overnight at 56 °C. Following that, 300 µL of protein precipitation solution (7 M Ammonium acetate, Tech & Innovation, Seongnam, Republic of Korea) was added, followed by centrifugation at 13,000 rpm for 15 min at 20 °C. Subsequently, 600 μL of the supernatant was transferred to a fresh 1.5 mL tube and 10 μL of RNase solution (10 mg/mL, G-spin Total DNA extraction Kit, iNtRON Biotechnology, Republic of Korea) was added. The mixture was then incubated at 65 °C for 1 h. Following this, 600 μL of the solution was added to the tube and subjected to centrifugation at 13,000 rpm and 20 °C for 15 min. The solution was divided equally into two 1.5 mL tubes, with 600 μL in each. Then, 600 μL of isopropanol was added to each tube, followed by gentle inversion and incubation at −20 °C for 1 h. The samples were then centrifuged at 13,000 rpm for 10 min at 4 °C. The supernatant was removed, and 300 μL of 70% ethanol was added to wash the DNA pellet. Finally, the sample was centrifuged at 13,000 rpm for 5 min at 4 °C, after which the supernatant was removed. The pellet was air-dried at room temperature for 1 h, and 50 μL of Tris-EDTA (TE) buffer (1× TE buffer, pH 8.0, Tech & Innovation, Republic of Korea) was added for storage.

#### 2.1.2. Polymerase Chain Reaction and Restricted Fragment Length Polymorphism

Information on HSP70 single nucleotide polymorphism (SNP) (polymorphic sites and localization within the gene) was retrieved from a previous study [9] and the SNP database of NCBI (https://www.ncbi.nlm.nih.gov/; M98823.1) (accessed on 10 September 2020). The HSP70 SNP was genotyped using a 5′-exonuclease activity (TaqMan) assay performed on an HT7900 system (Applied Biosystems, Carlsbad, CA, USA). SNP assay was obtained from Applied Biosystems as a Custom TaqMan SNP genotyping assay. The TaqMan SNP genotyping assay mix consisted of a forward primer, a reverse primer, and two probes: one probe perfectly matched the wild-type sequence variant and was labeled with VIC, while the other probe matched the mutant (SNP) sequence variant and was labeled with 6-carboxyfluorescein (FAM) (Table 1). The probes used a non-fluorescent quencher and a minor groove binder moiety (MGB), enabling the design of relatively short and more specific probe sequences. The TaqMan-MGB genotyping assay mix was provided at a 40 × concentration.

**Figure 2:**
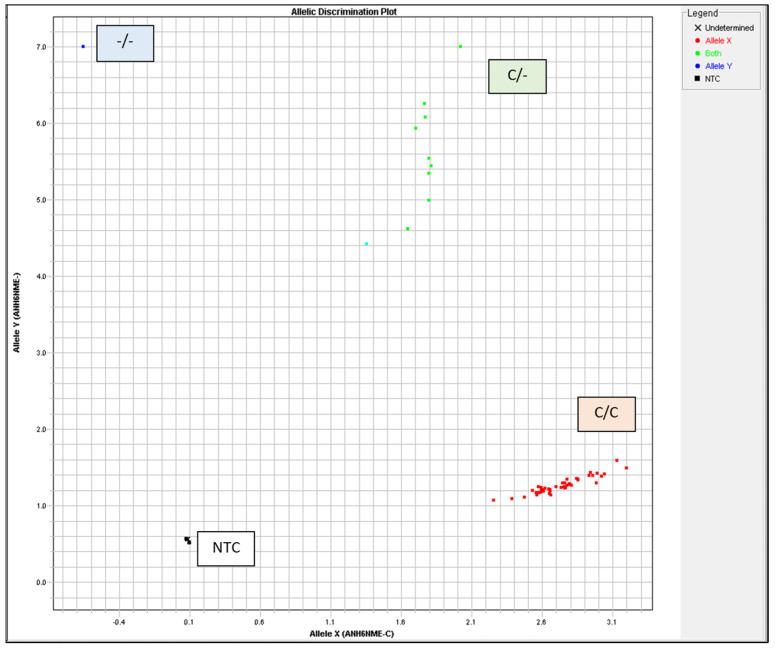
Heat shock protein 70 (HSP70) genotyping was visually inspected and validated by allelic discrimination plots. NTC, no template control.

The TaqMan™ GTXpress™ Master Mix (Applied Biosystems, USA) was used in a 5 µL total reaction volume with 20 ng DNA per reaction. Thermal Cylcler was used for PCR with the following conditions: 95 °C for 2 min, 40 cycles of 3 s at 95 °C, and 30 s at 60 °C. Allelic discrimination was automatically assessed using the ABI Prism HT7900 system (Applied Biosystems) with Sequence Detection System 2.4 software (Applied Biosystems). The autocaller confidence level was 95%. All measurements were visually examined and validated using allelic discrimination plots (cluster plots) (Figure 2, Table 2).

### 2.2. Experiment 1. Effects of Heat Stress and HSP70 Genotypes on Proliferation and HSP70 Gene Expression in the PBMCs of Beef and Dairy Calves

#### 2.2.1. Animals, Sampling, and Isolation of PBMCs

A total of 20 calves were selected among 60 calves based on HSP70 genotyping. PBMCs were isolated in 10 Korean native beef calves (age: 169.5 ± 3.32 days, with a BW of 166.9 ± 3.96 kg) and 10 dairy calves (age: 176.0 ± 4.90 days, with a BW of 178.2 ± 3.81 kg), with 5 replicates per HSP70 genotype (n = 5 for each group), including HSP70 CC- and C/-type beef calves (B-CC and B-C/-, respectively) and HSP70 CC- and C/-type dairy calves (D-CC and D-C/-, respectively). Blood was collected in early spring (March 2021) during a thermoneutral period when the temperature–humidity index (THI) remained consistently below 70, which is considered the upper critical threshold for cattle [15]. The isolation and culture of PBMCs were performed as described in our previous study [7]. Blood samples (20 mL) were drawn via jugular venipuncture, with sodium heparin (10 IU/mL) (BD Biosciences, Billerica, MA, USA) used as an anticoagulant to collect whole blood. Immediately after collection, blood samples were kept at room temperature and transported to the laboratory for separation and further analyses. For PBMC isolation, blood samples were processed within 8 h of collection. PBMCs were separated from whole blood using density gradient centrifugation. The whole blood was mixed with 1 × PBS (Hyclone, Laboratories, INC., Logan, UT, USA) in a 1:1 ratio and carefully layered over Histopaque-1077 (Sigma-Aldrich, Inc., St. Louis, MO, USA). All of the steps for PBMC isolation were carried out at room temperature following the manufacturer’s instructions. The isolated PBMCs were washed twice with 1 × PBS. The viability of the isolated PBMCs was determined using the Trypan Blue dye exclusion method [16]. The cell pellet was resuspended in serum-free RPMI medium (Sigma-Aldrich, Inc.) and cell numbers were determined using a hemocytometer (Neubauer-improved, Marienfeld, Germany) with 0.04% Trypan blue (Sigma Life Science, St. Louis, MO, USA). The viability of PBMCs typically exceeded 85% in calves. The PBMCs were resuspended at a concentration of 1 × 10^6^ viable cells/mL in RPMI 1640 medium (Sigma-Aldrich, Inc.) containing 25 mM HEPES (Sigma-Aldrich, Inc.) and supplemented with heat-inactivated fetal bovine serum (Sigma-Aldrich, Inc.), 2 mM L-glutamine (Sigma-Aldrich, Inc.), 100 U of penicillin (Sigma-Aldrich, Inc.), 100 µg of streptomycin (Sigma-Aldrich, Inc.), and 0.25 µg of amphotericin B/mL (Sigma-Aldrich, Inc.). The cells were cultured into 6-well plates. The time interval between blood collection and the establishment of cultures was kept under 8 h for each sampling period.

#### 2.2.2. Heat Stress Treatment

The PBMCs isolated from the 10 Korean male beef calves and 10 dairy calves were subjected to each treatment in triplicate for 48 h. All of the culture plates were initially incubated at 37 °C in a humidified CO_2_ incubator (5% CO_2_ and 95% air) for 48 h. After 48 h, the cells were exposed to either 37 °C continuously (Control; CON) or 42 °C (heat stress; HS) for 3 h. Following this, the cells were returned to the 37 °C incubator at 0, 1, 3, 6, and 12 h for further recovery analysis.

#### 2.2.3. Measurement of Cell Viability

Cell proliferation was assessed using the Cell Counting Kit-8 (CCK-8) (Dojindo Molecular Technology, Kumamoto, Japan). The cells were seeded at a density of 1 × 10^6^ viable cells/mL of medium in a 96-well plate. At the time following treatment (HS), 10 µL of CCK-8 solution was added to 90 µL of culture medium. The cells were then incubated for 3 h at 37 °C, and the optical density was measured at 450 nm using a microplate reader (Synergy 2, Biotek Instruments Inc., Santa Clara, CA, USA).

### 2.3. Experiment 2. Effects of Heat Stress and HSP70 Genotype on HSP70 Gene Expression in the Hair Follicles of Beef and Dairy Calves

**Figure 3:**
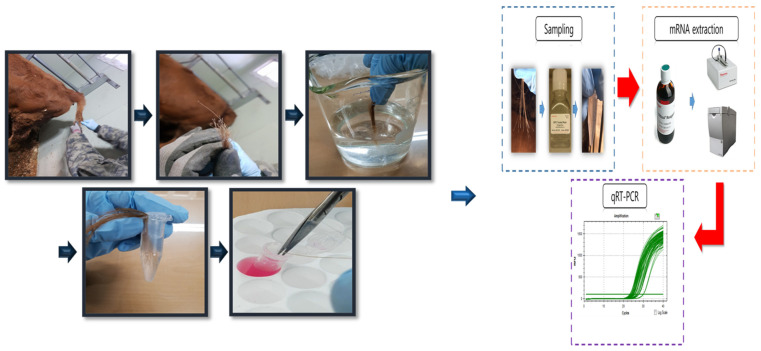
Hair follicle collection and storage methods for gene expression analysis.

A total of 20 calves were selected among 60 calves based on HSP70 genotyping. Hair follicle samples were collected from 10 Korean native beef calves (age: 169.5 ± 3.32 days, with a BW of 166.9 ± 3.96 kg) and 10 dairy calves (age: 176.0 ± 4.90 days, with a BW of 178.2 ± 3.81 kg), with 5 replicates per HSP70 genotype treatment (beef calves: B-CC and B-C/-; dairy calves: D-CC and D-C/-, n = 5 for each group). The hair follicle samplings were conducted as previously described by Kim et al. [13]. Hair follicles were collected from the tails of each calf at 14:00 h. The dates for sampling of hair follicles (9 Mar, 29 Mar, 19 Apr, 10 May, 31 May, and 21 June) were selected by referring to the weather forecast. The temperature and relative humidity (RH) inside and outside the barn were recorded at 1 h intervals using a sensor MHB-382SD (Lutron Electronic Enterprise, Taipei, Taiwan), and daily average (09:00 to 19:00 h) values of ambient temperature, RH, and the temperature–humidity index (THI) were calculated using the data presented in Table 3. The THI was calculated using the dry bulb temperature (Tdb, °C) and RH with the formula THI = (1.8 × Tdb + 32) − [(0.55 − 0.0055 × RH) × (1.8 × Tdb − 26.8)] as described in a previous study [17]. Figure 3 illustrates the progress of sampling hair follicles from tails. Approximately 25 to 30 strands of hair were collected by pulling them from the tail head of the calves. The hairs were firmly grasped as close to the skin as possible and then quickly pulled out. The hair follicles were washed with DEPC-treated water. The hair follicles were subsequently placed into a 5 mL specimen jar containing RNAlater™ (Ambion, Austin, TX, USA) for total RNA extraction. Total RNA was extracted from the hair follicles on the day of collection and stored at room temperature for 21 days in RNAlater™ (Ambion, Austin, TX, USA). The hair segments, including the hair follicle (1 cm from bottom), were cut and transferred into a 2 mL microcentrifuge tube containing 1 mL of TRIzol™.

### 2.4. Total RNA Extraction and Real-Time PCR Analysis

Total RNA was extracted from PBMCs and hair follicles using TRIzol™ reagent (Invitrogen, Carlsbad, CA, USA) following the manufacturer’s instructions [7]. The quality and quantity of the isolated RNA were assessed using an ND-1000 spectrophotometer (NanoDrop Technologies, Wilmington, DE, USA). The A260/280 ratio of all of the RNA samples was greater than 1.8, indicating a high purity. The RNA samples were stored at −70 °C until further analysis. First-strand cDNA was synthesized from 1 μg of RNA using the iScript cDNA synthesis kit (Bio Rad, Hercules, CA, USA). The expression of HSP70 gene in PBMCs and hair follicles was analyzed using real-time quantitative PCR (RT-qPCR) with SYBR- Green^®^ dye, following the method described previously [18]. All reactions were conducted in triplicate with a total reaction volume of 20 μL per well in a 96-well plate, using a Chromo4™ four-color real-time detector (MJ Research, Waltham, MA, USA). The reaction mixture consisted of 100 ng of cDNA, 10 μL of 2× SYBR Green PCR master mix (Bio-Rad), and 0.6 μL of 10 μM primers (Bioneer, Daejeon, Republic of Korea) in autoclaved water. The thermal cycling conditions included an initial incubation at 95 °C for 3 min, followed by 40 cycles of denaturation at 95 °C for 10 s, annealing at 60 °C for 30 s, and extension at 72 °C for 30 s. Subsequently, the samples were heated to 95 °C for 10 s, cooled to 65 °C for 5 s, and then gradually heated to 95 °C at a rate of 0.5 °C/s. The results were evaluated through post-PCR melt curve analysis of the amplification reactions, performed in triplicate from all samples by sequencing amplification products. Primers were designed using the National Center for Biotechnology Information Primer-BLAST (Table 4) [7]. The threshold cycles for each sample were normalized to housekeeping genes (GAPDH, RPS15A, and B2M) [19], and the relative expression of the target gene was quantified as the fold change of expression of the target gene relative to the expression of the thermoneutral control according to the 2^−ΔΔrCT^ method [20] (Table S1). The coefficient of variation for the housekeeping gene was verified prior to result calculation to ensure it remained below 5%.

### 2.5. Statistical Analysis

Data analysis was performed using JMP 5.0 software (SAS Institute Inc., Cary, NC, USA). In Experiment 1, the significance of differences in cell proliferation and HSP70 gene expression in PBMCs between HSP70 SNP groups (B-CC, B-C/-, D-CC, and D-C/-), recovery times (0, 1, 3, 6, and 12 h), and their interactions was determined using a two-way analysis of variance (ANOVA) followed by Tukey’s honestly significant difference (HSD) test. Differences in the rates of cell proliferation and the expression of HSP70 between CON and HS groups were evaluated using Student’s *t*-test. In Experiment 2, the significance of the differences in HSP70 gene expression in hair follicles among the HSP70 SNP groups (B-CC, B-C/-, D-CC, and D-C/-), sampling dates (9 March, 29 March, 19 April, 10 May, 31 May, and 21 June), and their interactions was determined using two-way ANOVA followed by the Tukey’s HSD test. Data are presented as the standard error of the mean. Significance was determined at an α level of 0.05, with tendencies specified at *p*-values between 0.05 and 0.10.

## 3. Results

### 3.1. Effects of Heat Stress and HSP70 Genotype on Cell Proliferation in PBMCs of Beef and Dairy Calves

**Figure 4:**
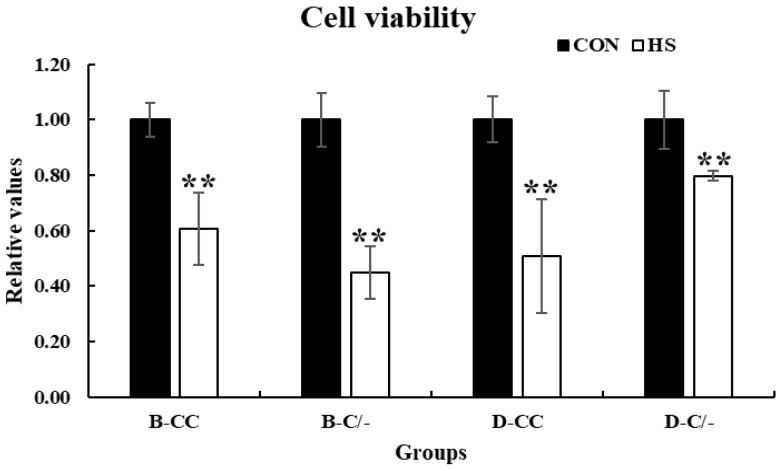
Effects of heat stress on peripheral blood mononuclear cell (PBMC) proliferation in beef and dairy calves. Cell viability was measured via Cell Counting Kit-8 (CCK-8) assay. Data are presented as means ± standard deviations (n = 5 for each group). ** Means with different superscripts differ significantly (*p* < 0.01) between control and heat stress groups based on Student’s *t*-test. B-CC, beef calves HSP70 CC type; B-C/-, beef calves HSP70 C/- type; D-CC, dairy calves HSP70 CC type; D-C/-, dairy calves HSP70 C/- type.

Cell viability was assessed using the CCK-8 assay to investigate the effects of HS on the proliferation of PBMCs. Cell proliferation significantly decreased (*p* < 0.01) in all HS-treated groups (B-CC, B-C/-, D-CC, and D-C/-) compared with that of the CON groups after 3 h of HS (Figure 4).

**Figure 5:**
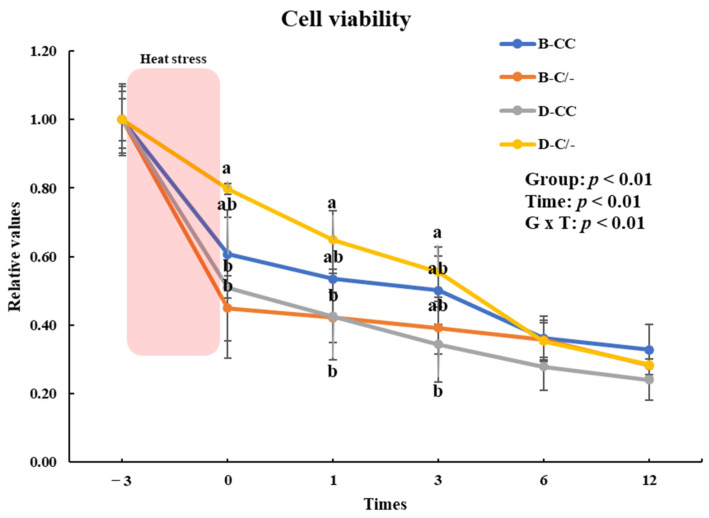
Effects of different heat shock protein 70 genotypes and times on cell proliferation in peripheral blood mononuclear cells (PBMCs) in beef and dairy calves. Cell viability was measured via Cell Counting Kit-8 (CCK-8) assay. Data are presented as means ± standard deviations (n = 5 for each group). *p*-values were determined using two-way ANOVA. ^a,b^ Means with different superscripts differ significantly (*p* < 0.05) within groups based on Tukey’s honestly significant difference (HSD) test. ANOVA, analysis of variance; B-CC, beef calves HSP70 CC type; B-C/-, beef calves HSP70 C/-type; D-CC, dairy calves HSP70 CC type; D-C/-, dairy calves HSP70 C/-type.

To evaluate the impact of the HSP70 genotype on cell proliferation, we investigated the changes in cell proliferation over time in the genotype groups (B-CC, B-C/-, D-CC, and D-C/-). An interaction between the time and treatment group was observed (*p* < 0.01) in the proliferation of PBMCs (Figure 5). The main effect of HSP70 SNP was altered cell proliferation (*p* < 0.01) (Figure 5). Cell proliferation was significantly higher (*p* < 0.05) in the D-C/-group than in the D-CC and B-C/-groups at 0 and 1 h following HS for 3 h (Figure 5). At 3 h after HS, a significant difference in cell proliferation was observed (*p* < 0.05) between the D-C/- and D-CC groups (Figure 5). Cell proliferation decreased (*p* < 0.01) with an increase in recovery time in all of the groups (Figure 5).

### 3.2. Effects of Heat Stress and HSP70 Genotype on HSP70 Gene Expression in PBMCs of Beef and Dairy Calves

**Figure 6:**
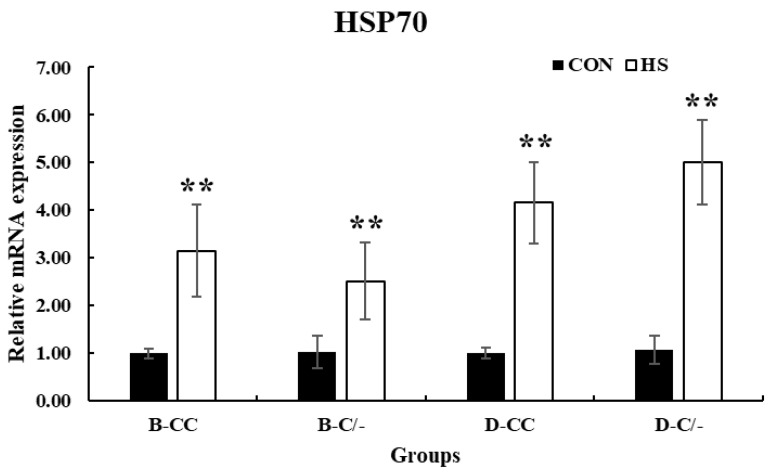
Effect of heat stress on heat shock protein 70 (HSP70) gene expression in beef and dairy calves. Data are presented as means ± standard deviations (n = 5 for each group). ** Means with different superscripts differ significantly (*p* < 0.01) between control (CON) and heat stress (HS) groups based on Student’s *t*-test. B-CC, beef calves HSP70 CC type; B-C/-, beef calves HSP70 C/-type; D-CC, dairy calves HSP70 CC type; D-C/-, dairy calves HSP70 C/-type.

The expression of HSP70 significantly increased (*p* < 0.01) in all HS-treated groups (B-CC, B-C/-, D-CC, and D-C/-) compared with that in the CON groups after 3 h of HS (Figure 6).

**Figure 7:**
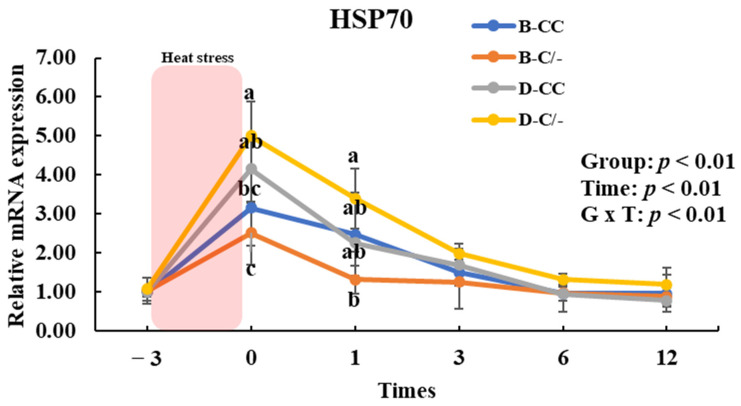
Effects of heat shock protein 70 (HSP70) genotypes and recovery times on HSP70 gene expression in peripheral blood mononuclear cells (PBMCs) in beef and dairy calves. Data are presented as means ± standard deviations (n = 5 for each group). *p*-values were determined using two-way ANOVA. ^a,b,c^ Means with different superscripts differ significantly (*p* < 0.05) within groups based on Tukey’s honestly significant difference (HSD) test. ANOVA, analysis of variance; B-CC, beef calves HSP70 CC type; B-C/-, beef calves HSP70 C/-type; D-CC, dairy calves HSP70 CC type; D-C/-, dairy calves HSP70 C/-type.

To assess the impact of HSP70 SNP on HSP70 gene expression, we analyzed the changes in HSP70 gene expression over time across the SNP groups (B-CC, B-C/-, D-CC, and D-C/-). An interaction between time and group was observed (*p* < 0.01) for HSP70 gene expression in the PBMCs (Figure 7). The main effect of the HSP70 SNP was altered (*p* < 0.01) HSP70 gene expression (Figure 7). The expression of HSP70 was significantly higher (*p* < 0.05) in the D-C/-group than in the B-CC and B-C/-groups at 0 h following HS for 3 h (Figure 7). In addition, HSP70 gene expression was significantly higher (*p* < 0.05) in the D-CC group than in the B-C/-group (Figure 7). At 1 h after HS, a significant difference in HSP70 gene expression was observed (*p* < 0.05) between the D-C/- and B-C/-groups (Figure 7). The time altered (*p* < 0.01) HSP70 gene expression for all groups (Figure 7).

### 3.3. Effects of Heat Stress and HSP70 Genotype on HSP70 Gene Expression in Hair Follicles of Beef and Dairy Calves

**Figure 8:**
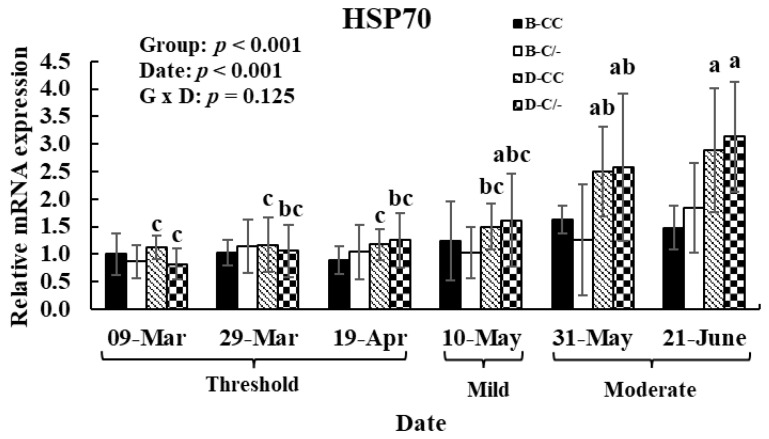
Gene expression level of heat shock protein 70 (HSP70) in hair follicles after exposure to various temperature–humidity index (THI) levels in beef and dairy calves. Data are presented as means ± standard deviations (n = 5 for each group). *p*-values were determined using two-way ANOVA. ^a,b,c^ Means with different superscripts differ significantly (*p* < 0.05) within groups based on Tukey’s honestly significant difference (HSD) test. ANOVA, analysis of variance; B-CC, beef calves HSP70 CC type; B-C/-, beef calves HSP70 C/-type; D-CC, dairy calves HSP70 CC type; D-C/-, dairy calves HSP70 C/-type.

To investigate the effects of HS and HSP70 SNP on HSP70 gene expression patterns after exposure to various THI levels, hair follicle samples were analyzed. The main effect of date (various THI levels) altered (*p* < 0.01) the HSP70 gene expression in hair follicles (Figure 8). In the D-CC and D-C/-groups, HSP70 gene expression was significantly higher (*p* < 0.01) in the moderate THI level group compared to the threshold and mild groups (Figure 8). However, no difference was observed in the B-CC and B-C/-groups in response to changes in THI levels. The main effect of HSP70 SNP altered (*p* < 0.01) the HSP70 gene expression in hair follicles (Figure 8). No interaction between the date and HSP70 SNP group was observed (*p* = 0.13) for HSP70 gene expression (Figure 8).

## 4. Discussion

Heat shock protein 70 (HSP70) plays a critical role in cellular defense mechanisms against heat stress (HS), ensuring protein homeostasis and protecting cells from thermal damage [7,13]. HSP70 polymorphisms, including HSP70-1, HSP70-2, HSP70-3, and HSP70-4, are localized on different chromosomes and play a role in heat resistance through their interactions with various genetic variants [21]. Among these, HSP70-1 and HSP70-2 show homology with HSPA1 and HSPA1L, which are situated on chromosome 6p21.3 [21]. Notably, HSP70A has been extensively studied for its role in cellular response, particularly in protecting cells from HS damage [9,22]. Genetic polymorphisms of the HSP70 gene, particularly single nucleotide polymorphisms (SNPs), impact its expression and functionality, affecting thermotolerance in cattle [9,11]. Previous studies have identified associations between HSP70 SNPs and various physiological traits, indicating that genetic variations in this protein may be used as markers to enhance heat resilience [10,23]. Considering the economic impact of HS on livestock productivity, elucidating the interplay between breed-specific differences and HSP70 polymorphisms is crucial for developing effective genetic selection and management strategies [4].

In this study, we investigated the effects of HS and genetic polymorphisms of HSP70 on cellular and molecular responses in beef and dairy calves, focusing specifically on peripheral blood mononuclear cells (PBMCs) and hair follicles. These findings reveal significant genotype- and breed-specific differences in cellular proliferation, HSP70 gene expression, and thermotolerance, providing insights into the adaptive mechanisms of thermal stress in cattle.

HS alters the morphology and membrane structure of PBMCs, reduces cell viability, and inhibits their proliferation [7,24]. The present study demonstrated that the reduction in PBMC proliferation across all groups under HS conditions indicates the negative impact of elevated temperatures on immune cell viability, even for the breed or HSP70 genotype. Proliferation rates varied significantly based on HSP70 genotypes and calf breeds during the recovery period following heat shock, with the dairy calves (C/-type) group exhibiting higher proliferation rates than those in the other SNP groups, particularly within the first 3 h of HS exposure. These results are consistent with those of studies in cattle that have shown reduced survival of PBMCs after heat shock during culture [25,26]. A previous study reported that HSP70 SNPs influence PBMC viability in cattle by altering the synthesis of HSP70 gene expression [27]. As a previous study reported [27], the presence of promoter variants may enhance the binding of the corresponding transcription factors. The overexpression of HSP70 indicates the activation of cellular protective mechanisms, which may contribute to improved cell viability. HSPs rapidly accumulate within cells when exposed to stressors, such as heat, hypoxia, metabolic stress, intense exercise, and infections [28], acting as molecular chaperones that help maintain cellular homeostasis and protect the cells from damage [8]. Among the various HSPs families, HSP70 plays a critical role in the HS response of immune cells [7]. HSP70 is required for developmentally regulated cell growth and proliferation. In the present study, HSP70 gene expression in PBMCs was significantly upregulated in response to HS, with marked variations observed across breeds and genotypes. The results obtained in this study revealed that dairy calves with the C/- genotype exhibited higher HSP70 gene expression, suggesting a potential genetic advantage in managing thermal stress. The upregulation of HSP70 gene expression in PBMCs following HS exposure marked its essential function as a molecular chaperone in facilitating stress adaptation [7]. The differences in HSP70 gene expression observed between genotypes and breeds reflect the impact of genetic and physiological factors on thermal response. Specifically, dairy calves showed a more significant increase in HSP70 gene expression compared to beef calves, likely reflecting their greater sensitivity to HS. These results suggest that specific HSP70 genotypes and different breeds provide a protective advantage by enhancing cellular repair mechanisms or reducing the adverse effects of HS through the activation of HSP70 synthesis.

The findings from this study reveal that HSP70 polymorphisms play a critical role in modulating the cellular response to HS in cattle, with distinct effects observed between breeds and genotypes. Variants in the HSP70 gene, particularly SNPs in regulatory and coding regions, are associated with differences in HSP70 expression and thermotolerance. For instance, the presence of the C/- genotype in Holstein dairy calves was linked to a higher expression of HSP70 in PBMCs, indicating a more robust cellular protective response compared to beef calves with the same genotype. This suggests that HSP70 polymorphisms may influence not only the inducibility of HSP70 expression but also the efficiency of protein stabilization and repair mechanisms during stress.

Concerns regarding the effects of HS and the need to better understand and mitigate their impacts are particularly relevant, especially for preventing economic losses. However, among previous studies on biological systems, only two have analyzed gene expression in surface samples. The results obtained in our previous study suggested that changes in HSP gene expression in the hair follicles of calves could be used as novel non-invasive biomarkers for HS [13]. Kim et al. [13] showed that HSP gene expression increases with high temperature–humidity index (THI) levels in both external environments and climatic control chambers. However, large-scale studies using surface samples, such as hair follicles, are needed to improve genetic tolerance, while considering animal welfare, facilitating collection, and minimizing additional stress. This current study showed that HSP70 gene expression in hair follicles increased with varying THI levels, particularly in dairy calves. Elevated HSP70 gene expression in dairy calves with the CC and C/- genotypes under moderate THI conditions indicates that these genetic variants may enhance the ability to respond effectively to thermal stress. In contrast, the lack of significant changes in beef calves suggests potential breed-specific differences in the dependence on HSP70-mediated pathways for thermoregulation in the body.

Thus, our results revealed interactions between HS conditions, breed differences, and HSP70 genetic polymorphisms in cattle. These findings demonstrate the critical role of HSP70 in modulating cellular and molecular responses to thermal challenges. Identifying thermotolerant genotypes presents opportunities for genetic selection and targeted management practices to enhance livestock resilience and productivity at increasing environmental temperatures. However, further studies are needed to explore the large-scale genetic and physiological networks that interact with HSP70 to deepen our understanding of thermotolerance in cattle.

## 5. Conclusions

The results obtained in this study suggest the critical role of genetic polymorphisms in HSP70 in regulating cellular and molecular responses to heat stress in cattle. The results revealed breed- and HSP70-genotype-specific variations in thermotolerance with implications for genetic selection and livestock management strategies. These results advance our understanding of heat stress adaptation in cattle by identifying HSP70 as a potential marker of heat resilience and establishing the utility of hair follicles as non-invasive tissue samples. However, this study has a limitation due to its lack of interaction with variant HSP70 polymorphisms in cattle. Therefore, further research is required to expand these findings by considering variant HSP70 polymorphisms and exploring their practical applications in addressing the ensuing challenges in the beef and dairy industry in the changing global climate.

## Figures and Tables

**Table 1:** List of primers and probe sequences used for single nucleotide polymorphism (SNP) assay.

Gene	Accession Number	Primer/Probe	Sequence (5′–3′)
HSP70	M98823.1	Forward	CCAGGGCGCTGATTGGTT
		Reverse	GCCAGGTCGGGAATATTCCA
		Probe 1	CCCCTGGCTTTCT
		Probe 2	CCCCCTGCTTTCT

HSP70 = heat shock protein 70.

**Table 2:** Genotype frequency of heat shock protein 70 (HSP70) polymorphisms in beef and dairy calves.

Genotypes	Allele Frequencies (%)	Beef Calves (n)	Dairy Calves (n)
Total (n = 60)	100	30	30
C/C (n = 44)	73.3	21	23
C/- (n =16)	26.7	9	7

**Table 3:** Mean ambient temperature, (%) relative humidity, and temperature–humidity index (THI) on the selected sampling dates from March 09 to June 21 during Experiment 2.

Variables	Date					
	Threshold			Mild	Moderate	
	9 March	29 March	19 April	10 May	31 May	21 June
Temperature (°C)	13.1	16.3	21.1	24.1	29.9	30.1
Relative humidity (%)	61.4	50.4	58	84.5	77.5	80.2
THI	56.3	60.6	67.4	74.0	82.5	83.2

THI = temperature–humidity index.

**Table 4:** Primer sequences, lengths, and accession numbers.

Gene	Accession Number	Sequence	Length (bp)
HSP70	U09861	F: TACGTGGCCTTCACCGATAC R: GTCGTTGATGACGCGGAAAG	171
GAPDH	NM_001034034.2	F: GGCAAGGTCATCCCTGAG R: GCAGGTCAGATCCACAACAG	166
RPS15A	NM_001037443.2	F: CCGTGCTCCAAAGTCATCGT R: GGGAGCAGGTTATTCTGCCA	200
B2M	NM_173893.3	F: GACACCCACCAGAAGATGGA R: CAGGTCTGACTGCTCCGATT	125

HSP70 = heat shock protein 70; GAPDH = glyceraldehyde-3-phosphate dehydrogenase; RPS15A = ribosomal protein S15a; B2M = beta-2-microlobulin.

## Data Availability

The data presented in this study are available in this paper.

## Supplementary Materials

The following supporting information can be downloaded at: https://www.mdpi.com/article/10.3390/ani15040475/s1, Table S1: qPCR gene expression in cells and hair follicles.
